# Therapeutic pulmonary telerehabilitation protocol for patients affected by COVID-19, confined to their homes: study protocol for a randomized controlled trial

**DOI:** 10.1186/s13063-020-04494-w

**Published:** 2020-06-29

**Authors:** Juan Jose Gonzalez-Gerez, Carlos Bernal-Utrera, Ernesto Anarte-Lazo, Jose Antonio Garcia-Vidal, Jose Martin Botella-Rico, Cleofas Rodriguez-Blanco

**Affiliations:** 1 Fisiosur I+D Research Institute, Garrucha, Almería Spain; 2 Deparment Nursing, Physiotherapy and Medicine, University of Almeria, Almeria, Spain; 3 Doctoral Program in Health Sciences, University of Seville, Seville, Spain; 4 Centre of Precision Rehabilitation for Spinal Pain (CPR Spine), School of Sport, Exercise and Rehabilitation Sciences, University of Birmingham, Birmingham, UK; 5 Physiotherapy Department, University of Murcia, Murcia, Spain; 6 Physiotherapy Department, University Cardenal Herrera-CEU, Elche, Alicante Spain; 7 Physiotherapy Department, University of Seville, Seville, Spain

**Keywords:** Pandemics, Coronavirus infections, Physical therapy specialty, Telerehabilitation, Exercise therapy, Pulmonary ventilation, Randomized controlled trial, COVID-19

## Abstract

**Background:**

In December 2019, 27 cases of pneumonia, of unknown cause, were identified in the province of Hubei (China). The WHO declared the situation as a Public Health Emergency of International Concern, and it was finally declared a global pandemic on March 11, 2020. The Spanish Government obliges the entire population to remain confined to their homes, with the exception of essential basic services, to stop the spread of COVID-19. Home isolation implies a notable physical deconditioning. Telerehabilitation methods have reported positive experiences, and we propose to study in affected patients of COVID-19, due to the general house confinement of the entire Spanish population.

**Methods:**

Patients will be recruited in the regions of Andalusia, Murcia, and Valencia (Spain). Patients will remain confined to their homes, and there, they will carry out their assigned exercise program, which will be controlled telematically. Evaluators will attend to carry out all measurements at the beginning, during, and end of the study, telematically controlled. The patients will be randomly divided into three groups, two of them will perform a home exercise program (breathing exercises or non-specific exercises for muscle toning) and the third group will perform sedentary activities, using mental activation techniques, and will act as a sham group. We will evaluate respiratory variables and other variables of the physical state through physical tests, effort, and perceived fatigue. The data will be statistically analyzed, and the hypotheses will be tested between the groups, using the SPSS software, v.24, considering a 95% confidence interval.

**Discussion:**

We will analyze the results, in terms of the level of fatigue and perceived exertion, physical health, and maintenance of respiratory activity of two types of exercise programs, toning and respiratory, applied in patients affected by COVID-19 during the period of home confinement. We intend to investigate a field not previously studied, such as the repercussion of carrying out a toning and respiratory exercise program in these patients, in historical circumstances that no one had previously observed in Spain, since the general population has never been forced to remain confined in their homes, due to a pandemic infection, by a coronavirus (COVID-19). Observing the effects that these two home exercise programs could produce in patients infected with COVID-19, we will try to better analyze and understand the mechanisms that are associated with the worsening of breathing in this type of patient.

**Trial registration:**

Brazilian Clinical Trial Registry RBR-6m69fc. Registered on March 31, 2020.

## Background

In December 2019, 27 cases of pneumonia, of unknown cause, were identified in Hubei (China) [1]. On January 7, 2020, the Chinese authorities identified a new virus, which was called SARS-CoV-2, and since then, it is commonly known as COVID-19 [2]. Subsequently, on January 30, 2020, the WHO declared the situation as a Public Health Emergency of International Concern [3], and it was finally stated a global pandemic on March 11, 2020 [4]. Since March 14, 2020, the Spanish Government decreed the state of alarm, which implied house confinement for the entire population, with the exception of essential basic services, to stop the spreading of COVID-19 [5].

This virus is characterized by its contagion capacity, which, as it has been established, can occur through three routes: the respiratory route, by direct contact, and through feces (although more research is needed in this route) [6]. It has been estimated that the incubation period is 5.2 days for a 95% confidence interval (CI) [4.1–7.0], and the basic number of reproduction R0 is 2.2 for a 95% CI [1.4–3.9] [7].

As of May 24, 2020, countries that are considered the most affected are the USA (1,568,448 cases; 94,011 deceased), the UK (257,158 cases; 36,675 deceased), Italy (229,327 cases; 32,735 deceased), Brazil (330,890 cases; 21,048 deceased), and Spain (235,290 cases; 28,678 deceased), being according to the WHO, the total of 5.206.614 confirmed cases and 337.736 confirmed deaths by COVID-19, presenting cases in 216 countries or territories [7, 8]. The main signs and symptoms described by recent studies [9–11] are fever (98%), cough (76%), and myalgia or fatigue (44%); atypical symptoms have also been identified: sputum (28%), headache (8%), hemoptysis (5%), vomiting (5%), diarrhea (3%), and dyspnea in approximately half of the patients. In addition to lymphopenia, identified in 63% of cases, pneumonia is present in all patients. Complications include acute respiratory distress syndrome (29%), acute heart injury (12%), and secondary infections (10%). In addition, other symptoms more striking such as chemosensory dysfunction have also been described [12]. Some patients had at least one underlying disease (such as hypertension, chronic obstructive pulmonary disease), and many of them require treatment in intensive care units (ICU) [9–11].

It has been estimated that 80% of the patients will present mild symptoms (without hospital admission). The remaining 20% will need medical care, and 5% of them will require admission to the ICU [10, 13]. The average time from onset of symptoms to recovery is 2 weeks when the disease has been mild and 3–6 weeks when the disease has been severe or critical [14]. The WHO recommends, for 80% of patients who do not require hospital admission, the need for very restrictive home isolation and confinement in individual rooms at home to avoid the spreading of the virus [15]. The total isolation of these patients requires non-face-to-face medical attendance, performing telematic control to monitor the evolution of the patient affected by COVID-19.

Home isolation implies a notable physical deconditioning, not only at the musculoskeletal level, but also implies negative metabolic changes [16, 17]. It could trigger peaks in type II diabetes, which could lead to worsening of the clinical picture in patients affected by COVID-19 [18] and repercussions on emotional state [19]. Physical activity programs have reported beneficial effects in maintaining muscle mass and strength [20] and prevents metabolic and nutritional decompensations caused by inactivity [21]. The implementation of a physical activity program in patients with mild symptoms of COVID-19 could achieve breathing benefits, reducing the rate of aggravation and hospital admissions in these patients.

Our study aims to verify and validate the efficacy of a telerehabilitation program, through therapeutic exercise at the respiratory level, and the maintenance of vertebral and thoracic muscle tone, in patients affected by COVID-19. There is evidence on the efficacy of domiciliary exercise-based treatments in patients with respiratory disorders, and based on this, this could be the therapeutic method of choice to allow the treatment and supervision of patients affected by COVID-19 during home confinement [22–24]. Telerehabilitation has begun to be implemented in other rehabilitation fields, such as cardiac rehabilitation, cancer rehabilitation, neurological rehabilitation, and spinal cord injuries [25–27]. Some studies have pointed out the effectiveness of these methods [28, 29]; however, the last systematic reviews and meta-analyses highlight the limited evidence, mainly due to the lack of high-quality research studies [30, 31]. In general terms, telerehabilitation methods have been reported as positive experiences by patients [32] and can represent an important way to reduce associated healthcare costs [30]. Nonetheless, in this case, the reasons for implementing telerehabilitation are not economic, but of necessity, due to the general house confinement of the entire Spanish population established to avoid the spreading of the virus.

## Objectives

### Primary objective

The aim of our study is to analyze the respiratory effects of a therapeutic exercise program in patients affected by the coronavirus (COVID-19), during the period of home confinement.

### Secondary objective

The secondary objective is to compare the respiratory effects obtained among patients affected by the coronavirus (COVID-19), after the application of a respiratory exercise program (REP), compared to those who perform a non-specific toning exercise program (NTEP) and a to sedentary control group performing a sham program, during the period of house confinement.

## Methods/design

### Trial design

The trial design is a randomized, controlled, parallel, double-blind, three-arm clinical trial of treatment.

### Sample selection

Patients will be recruited through a text message transmitted on social network (WhatsApp); they will be contacted with a general message informing of the possibility of participating in a physiotherapy study; all those interested will be informed later in greater detail, in the regions of Andalusia, Murcia, and Valencia (Spain). Therefore, any patient resident in the autonomous communities of Andalusia, Murcia, and Valencia may participate in this research. The messages will be sent to positive cases through PCR tests (polymerase chain reaction) classified by the epidemiology services of each region. In terms of privacy, only patients who have validated their contact for information and research services to the corresponding epidemiology services will be contacted. Later, they will be selected according to the listed eligibility criteria. The study will take place at the selected patients’ home, where the evaluators will attend to carry out all measurements at the beginning, during, and end of the study. All measurements will be instructed and telematically controlled by the study evaluator, who will provide patients the necessary assessment materials, which are described below (the “Outcome measurements” section).

Only mild cases will be recruited, so all patients must be confined at home. We will exclude those patients who required derivation to hospital care. The criteria are based on those published by the Spanish Society of Family and Community Medicine (SEMFYC) and can be checked on the “Exclusion criteria” section [33].

### Inclusion criteria

The following are the inclusion criteria:

- Age 18–75 years
- Patients with positive polymerase chain reaction (PCR) test
- Patients who are affected by the coronavirus (COVID-19) and are in home confinement

### Exclusion criteria

The following are the exclusion criteria:

1. Patients with chronic lung conditions
2. Patients with chronic kidney disease
3. Patients affected with chronic neurological disorders
4. Patients suffering from hypertension and cardiovascular conditions without medical treatment
5. Patients affected with grade III osteoporosis
6. Patients affected with acute phase of rheumatologic disorders
7. Patients affected with acute phase of disc abnormalities
8. Patients who have had respiratory conditions in the last 12 months
9. Patients who have recent musculoskeletal disorders and who are not fully recovered from their injuries
10. Patients who have received physical therapy treatment in the last 3 months
11. Patients affected with chronic mental and/or psychological disturbances
12. Red flags for serious conditions (night pain, severe muscle spasm, loss of involuntary weight, symptom mismatch)
13. Patients classified as moderate/severe cases based on the following criteria [33]:
  - Respiratory rate ≥ 30 brpm
  - SpO2 < 92%
  - Cardiac rate > 125 bpm
  - Hypotension (SBP < 90 mmHg or DBP < 60 mmHg)
  - Severe dyspnea (minimal effort or rest)
  - Signs of respiratory compromise (cyanosis, use of accessory muscles)
  - Hemoptysis
  - Altered alertness (lethargy, acute confusion, disorientation)
  - Inability to orally intake due to unintended vomiting or a significant number of bowel movements (≥ 10 per day) suggesting dehydration or hydroelectric disturbances
  - Significant impact on the general condition
  - High clinical suspicion of pneumonia requiring radiography, based on worsening of dyspnea, more than 7 days with fever, respiratory rate higher than 22 brpm, and alteration in auscultation. Punto 3.

### Interventions

Patients will perform exclusively assigned therapeutic exercises or sedentary activities (depending on randomized allocation to the study groups) and may not combine with other physical therapy or sports physical activity. Any interference in the treatment will be grounds for exclusion, participants will be asked in the daily contacts if they have carried out any activity that can be considered as meddling in the treatment. If participants are required to combine the intervention with medications, it will be registered.

Exercise monitoring will be developed through telerehabilitation tools, that is, emerging technology through which medical rehabilitation care can be provided through a distance. Patients will be encouraged to carry out treatment and follow-up completely, through videoconferences that will encourage them to improve their health status through their personal effort and will reduce the rate of loss to follow-ups and dropouts. In the event that losses to follow-up or dropouts of more than 15% are observed, we will perform an intention-to-treat analysis.

### Group 1: Respiratory exercise program

The REP will consist of 10 exercises based on the recommendations made by the official physiotherapy organizations at the institutional level (College of Physiotherapists of the Community of Madrid, Spain) [34]; with scientific evidence, the method of active cycle of breathing techniques uses an alternate depth of breathing to move mucus from small airways at the bottom of the lungs to bigger airways, where they can be cleaned in an easier way by coughing. In addition, postural changes can modify positively the ventilation/perfusion index [35]. The REP will be taught to patients telematically in the first session, and it will be carried out once a day, for 21 days, at the patient’s home, between 11 a.m. and 12 a.m., in the morning, and is described in Additional file 1. The REP will be reinforced by a physical therapist daily, through telematic control by videoconference with each patient.

### Group 2: Non-specific toning exercise program

The NTEP will consist of 10 exercises based on the recommendations about toning exercises, made by the official physiotherapy organizations at the institutional level (College of Physiotherapists of the Community of Andalusia, Spain) [34]; with scientific evidence, it has been demonstrated that exercise therapy produces many benefits in the immune/defense system. Due to the established relationship between this system and COVID-19 effects, we have decided to include a group of unspecific exercises and to analyze how patients will benefit from this kind of exercises [36, 37]. We also try to avoid physical deconditioning, with the physiological deterioration it implies [16, 17].

The NTEP will be taught to patients in the first session, and it will be carried out once a day, for 21 days, at the patient’s home, between 11 a.m. and 12 a.m., in the morning, and is described in Additional file 1. The NTEP will be reinforced by a physical therapist daily, through telematic control by videoconference with each patient.

### Group 3: Sham program

The sham program consists of 10 sedentary exercises based on sophrology and meditation, with mental exercises of visualization, concentration, and mental activity. The program will not follow a linear progression nor will it have the objective of obtaining benefits through meditation; it will only try to interact with the subjects for an hour. The sham program (SP) will be taught to patients in the first session telematically, and it will be carried out once a day, for 21 days, at the patient’s home, between 11 a.m. and 12 a.m., in the morning. The SP will be reinforced by a physical therapist not specialized in meditation daily, through telematic control by videoconference with each patient. After the study, patients in the SP group will carry out the exercise program that has shown the greatest benefits in the state of health (REP or NTEP), which we consider ethically necessary.

### Procedure for adverse effects

Participants will be daily assessed through a phone call by a member of the study team, who will ask for possible adverse events. This follow-up will be performed through a checklist translated and adapted from “Criteria for clinical evaluation during telephone follow-up of home care” published by SEMFYC [33], and it can be checked in Additional file 2. In addition, a telephone number will be provided to the participants. If any complication or doubt, they should contact the member of the study team, and in the function of episode features, they will be informed of the procedure to follow.

The Trial Steering Committee is made up of three researchers external to the research group; they will meet weekly and analyze the ongoing results of the research; they have the power to partially or completely paralyze the course of the study. This study does not contemplate any intervention of public organisms for its development.

### Outcome measures

The data will be collected by personnel attached to the research group who have previously been instructed in the procedures to follow and do not know the group to which the patients belong; the captures sent by the patients will be stored and classified; the evaluators will transfer the numerical values to an excel table. The images sent by the patients and the excel table are encrypted, and only the evaluators and the main researchers have access to it. This information is updated through a security cloud. All outcomes will be measured daily using WhatsApp or by email for 21 days.

- Visual Analog Scale Fatigue (VASF), for fatigue measurement [38]. Patients participating in the study will indicate the intensity of their fatigue by means of the VAS through the smartphone application called “Visual Scale” (Apple Store and Google Play). They will have to signal in a horizontal line where they would place their fatigue, being 0 “no fatigue” and the 10 would be “the worst imaginable fatigue”. VASF will be controlled telematically by the evaluators through the Smartphone application. The evaluator will ask the patient to indicate their level of VASF in the “Visual Scale” application and to take a screenshot of the smartphone to obtain the established value for VASF, which is calculated as the average value of two attempts. The patient must send the VASF screenshot daily to the evaluator, via WhatsApp or email.
- Forced expiratory volume in 1 s (FEV1). The Piko-1 spirometer device will be sent to the home of each patient by post, and a telematic control of its use will be carried out. The evaluator will ask the patient to indicate their level of FEV1 assessed by the Piko-1 device, twice each time. FEV1 obtained values by patients must be sent to the evaluator, via WhatsApp or email. We will calculate the means of each 2 measurements, assessed by patients every day [39, 40].
- Peak expiratory flow (PEF). The evaluator will ask the patient to indicate their level of PEF assessed by the Piko-1 device, twice each time. After obtaining the values, patients will send them to the evaluator, via WhatsApp or email. We will calculate the means of each 2 measurements, assessed by patients every day [41].
- Six-Minute Walk Test (6MWT). The 6MWT will be performed by all patients in their home, under the telematically supervision of a physiotherapist. All patients will receive the same instructions before the walk and will be encouraged by the physiotherapist who repeated set phrases every minute during the walk. The distance covered during the test will be recorded in meters telematically by the patient’s smartphone. Patients will be allowed to stop and rest during the test, but they will be instructed to resume walking as soon as they felt able to do so. Patients will send the results of the 6MWT screenshot daily to the evaluator, via WhatsApp or email [42].
- Thirty Seconds Sit-To-Stand Test (30STST). Evaluators will ask patients to place a straight-backed armless chair, with a hard seat, which will be stabilized by placing it against a wall, considering floor to seat height will be between 45 and 50 cm. Seated participants will be asked to come forward on the seat until their feet will be flat on the floor and to fold their upper limbs across the chest, without moving it during all tests. Patients will be then instructed to stand up all the way and sit down once without using the upper limbs. Patients will start in the seated position on the chair and, upon command telematically, they will stand up, and then they will return to sitting as many times as they could, in a 30-s time period [41–43]. The evaluators will control this test telematically by videoconference.
- Multidimensional Dysphnoea-12 (MD12) Spanish version [44]. The MD12 questionnaire will be self-administered and will be performed at the end of the 6MWT. Patients will send the results of the MD12 screenshot daily to the evaluator, via WhatsApp or email.
- Borg Scale (BS). The Borg Scale, of perceived effort, measures the entire range of effort that the individual perceives when exercising. This scale gives criteria to make adjustments to the intensity of exercise, that is, to the workload, and thus forecast and dictate the different intensities of exercise in sports and medical rehabilitation. The BS will be completed by patients at the beginning of each session and after completing the exercise program, as well as at the end of the tests, every day (6MWT, 30STST). Patients will send the results of the BS screenshot daily to the evaluator, via WhatsApp or email [45, 46].

### Participants’ timeline

**Fig. 1:**
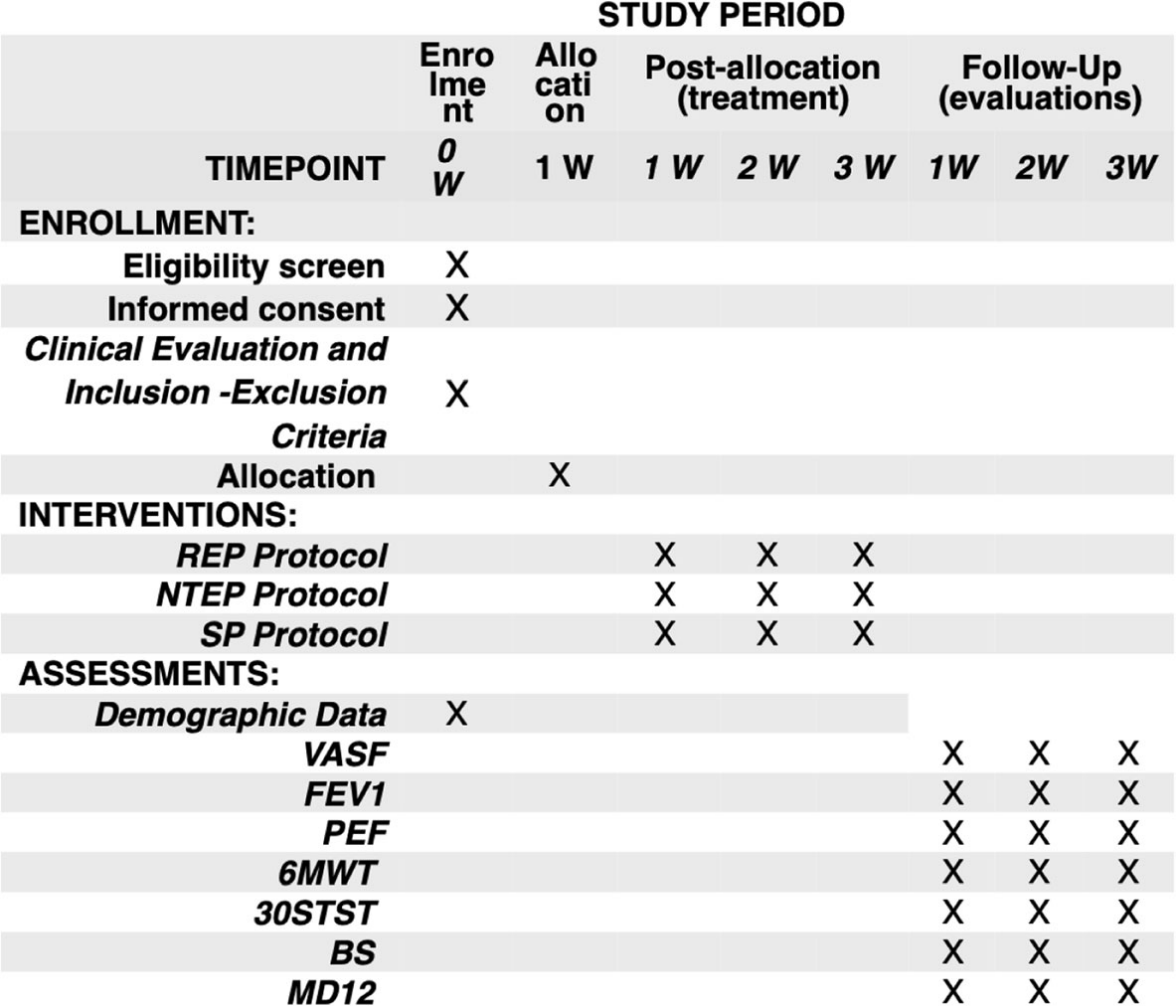
SPIRIT flow diagram. W, week; REP, respiratory exercise program; NTEP, non-specific tonic exercise program; SP, sham group; VASF, visual analog scale fatigue; FEV1, forced expiratory volume in 1 s; PEF, peak expiratory flow; 6MWT, Six-Minute Walk Test; 30STST, Thirty Seconds Sit-To-Stand Test; BS, Borg Scale; MD12, Multidimensional Dysphnoea-12

A brief Standard Protocol Items: Recommendations for Interventional Trials (SPIRIT) flow diagram is provided in Fig. 1, and a populated SPIRIT checklist is provided in Additional file 3.

### Sampling and sample size calculation

The sampling of our research will be non-probabilistic, since we will contact the patients through social networks, and not the entire population belongs to these networks, so the selection of the sample will be possible only among those who voluntarily respond to our request.

The sample size will be calculated using the Granmo calculator v.7.12, based on the minimum clinically important differences in VASF [43], and estimating an alpha risk of 5% (0.05), a beta risk of 10% (0.10), in a unilateral contrast, a typical deviation of 12.5% (0.125), a minimum difference to detect of 10% (0.10), and a rate of follow-up losses of 15%, for which 32 subjects are required in each group, assuming that there are three groups. If the rate of loss to follow-up were observed greater than 15%, we will perform an intention-to-treat analysis. Finally, we will include 114 patients who will be divided into three groups, each group of at least 38 subjects, being able to overcome this value to assume the possible loss of follow-up.

### Randomization

Patients will be divided into three groups by means of balanced randomization, carried out with free software (http://www.randomized.org/). The randomization sequence will only be performed by the principal investigator and auditor. No participant in the study will have access to the randomization sequence, which will be hidden and saved, to guarantee a correct randomization with security.

### Blinding

Evaluator and patients in the study will be blinded during the entire process. The evaluator will be unaware of the study objectives and the randomized distribution of patients to study groups and will not have access to the randomization sequence. Meanwhile, although blinding for patients will not be possible to achieve it completely, subjects will be unaware of other treatment modalities, and they will not know if they belong to the intervention or sham group.

### Statistical analysis

The statistical analysis will be carried out through the IBM-SPSS Statistics 24 software, considering the Kolmogorov-Smirnov test as the standard normality test. We will analyze the intragroup hypothesis contrast inference by Student’s *t* test for paired variables with parametric distributions and Kruskal-Wallis *H* for non-parametric distributions. We will analyze the intragroup hypothesis contrast inference by one-factor ANOVA in the case of parametric distributions and Kruskal-Wallis *H* for non-parametric distributions. We will apply a posteriori analysis (post hoc) through Bonferroni’s contrast for parametric distributions and Mann-Whitney’s *U* for non-parametric ones. The confidence level used will be 95% (0′05), and the power of the study will be 90% (0′1). If the rate of loss to follow-up were observed greater than 15%, we will perform an intention-to-treat analysis.

A sub-analysis will be carried out, depending on the age of the patients, sex, and autonomous community of residence.

## Discussion

This article presents a detailed description of a randomized controlled trial designed to analyze the results in terms of the level of fatigue and perceived exertion, physical health, and maintenance of respiratory activity of two types of exercise programs, toning and respiratory, applied in patients affected by COVID-19 during the period of home confinement. We intend to investigate a field not previously studied: the repercussion of carrying out a toning and respiratory exercise program in these patients, in historical circumstances that no one had previously observed in Spain, except the Spanish flu of 1918 where scientific knowledge and intervention capacity were very limited, since the general population had never been forced to remain confined at home due to a pandemic infection. Because some of these patients develop severe symptoms associated with respiratory distress, we consider that this study could be of interest in public health, to prevent worsening of the respiratory status of these patients, and so that we could understand the mechanisms that produce these serious alterations, which in many cases require hospitalization and the use of mechanical ventilation. We propose two types of treatments, one using breathing exercises exclusively and the other based on non-specific muscle toning exercise.

Measuring the effects that these two home exercise programs could produce in patients infected with COVID-19, we will try to better analyze and understand the mechanisms that are associated with worsening of breathing in this type of patient. Our results seek to analyze whether respiratory muscle stimulation, or vertebral/thoracic tonic musculature, is a protective factor in maintaining the health of these patients, and if together with cardiovascular stimulation they could have a specific influence on the prevention of severe respiratory disturbances suffered by some of these patients.

We have designed a randomized, controlled, double-blinded clinical trial, with the aim of contributing to increasing scientific knowledge on this matter, so new lines of future research could be developed.

The results will be communicated to the Spanish health authorities internally and to other relevant groups through publications in medical journals.

### Trial status

This is the second and definitive protocol version. Participants will be recruited between March 31, 2020, and April 30, 2020. Study completion is expected to be June 2020. The study protocol has been submitted before the end of the recruitment and before the last patient.

## Supplementary information

**Additional file 1.** Appendix 1. Respiratory Exercise Program and Non-specific Toning Exercise Program.

**Additional file 2.** Appendix 2. Checklist for telephone monitoring.

**Additional file 3.** SPIRIT 2013 Checklist: Recommended items to address in a clinical trial protocol and related document.

## Data Availability

The datasets analyzed during the current study are available from the corresponding author on reasonable request. The data will be available after the main publication of them; for other circumstances, they should consult the corresponding author. Any data required to support the protocol can be supplied on request.

## Abbreviations

SPIRIT: Standard Protocol Items Recommendations for Interventional Trials
REP: Respiratory exercise program
NTEP: Non-specific tonic exercise program
SP: Sham group
VASF: Visual Analogue Scale Fatigue
FEV1: Forced expiratory volume in 1 s
PEF: Peak expiratory flow
6MWT: Six-Minute Walk Test
30STST: Thirty Seconds Sit-To-Stand Test
BS: Borg Scale
MD12: Multidimensional Dysphnoea-12
