# Supplementary material for: Therapeutic pulmonary telerehabilitation protocol for patients affected by COVID-19, confined to their homes: study protocol for a randomized controlled trial
Source: Trials. 2020 Jun 29;21:588. doi: 10.1186/s13063-020-04494-w (PMC7322707; doi:10.1186/s13063-020-04494-w)
Supplement: Supplementary file 1 — Additional file 1. Appendix 1. Respiratory Exercise Program and Non-specific Toning Exercise Program. [file 13063_2020_4494_MOESM1_ESM.pdf]

Respiratory Exercise Program – COVID19

- EXERCISE 1. Reeducation of the posterior region of the diaphragm

The patient will be layed in supine position over a hard surface with a pillow under head and knees.First, the patient will perform an expiration with pursed lips until produce an abdominal wall contraction. Later, the patient will perform a deep nasal inspiration with the mouth closed. With this maneuver, the aim is to bring the air to the belly; when plenty of aire, the aire will be expulsed while the abdomen is contracted. 10-15 repetitions in the morning and 10-15 in the evening.

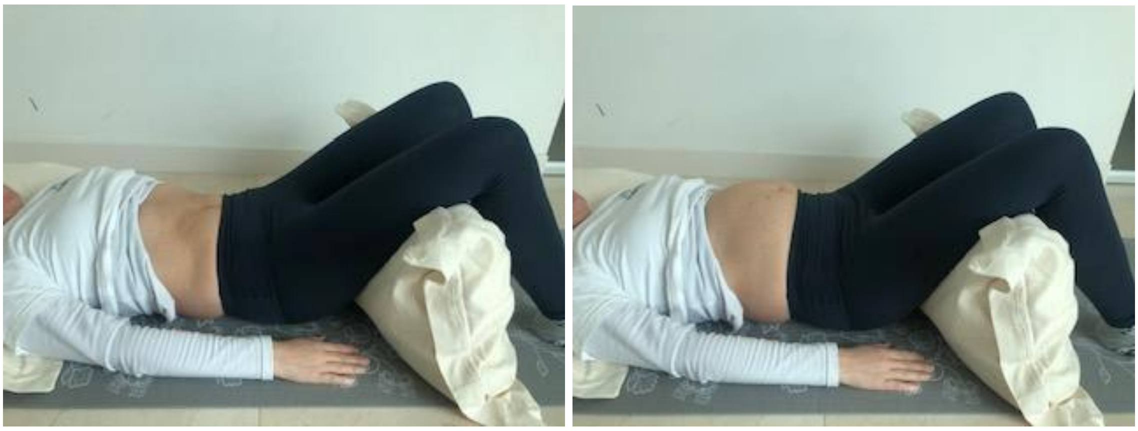

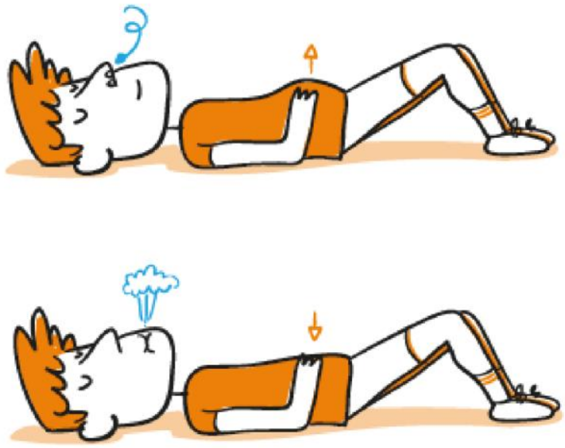

Picture from College of Physical Therapists, Community of Madrid

- EXERCISE 2. Reeducation of right hemidiaphragm

The patient will be layed in lateral decubite over right side, with a pillow under thoracic wall. Both low limbs will be semiflexioned and upper limbs in a confortable position. At first, the patient will perform an expiration with pursed lips until produce an abdominal wall contraction. Later, the patient will perform a deep nasal inspiration with the mouth closed. With this maneuver, the aim is to bring the air to the belly; when plenty of aire, the aire will be expulsed while the abdomen is contracted. 10-15 repetitions in the morning and 10-15 in the evening.

- EXERCISE 3. Reeducation of left hemidiaphragm

The patient will be layed in lateral decubite over left side, with a pillow under thoracic wall. Both low limbs will be semiflexioned and upper limbs in a confortable position. At first, the patient will perform an expiration with pursed lips until produce an abdominal wall contraction. Later, the patient will perform a deep nasal inspiration with the mouth closed. With this maneuver, the aim is to bring the air to the belly; when plenty of aire, the aire will be expulsed while the abdomen is contracted. 10-15 repetitions in the morning and 10-15 in the evening.

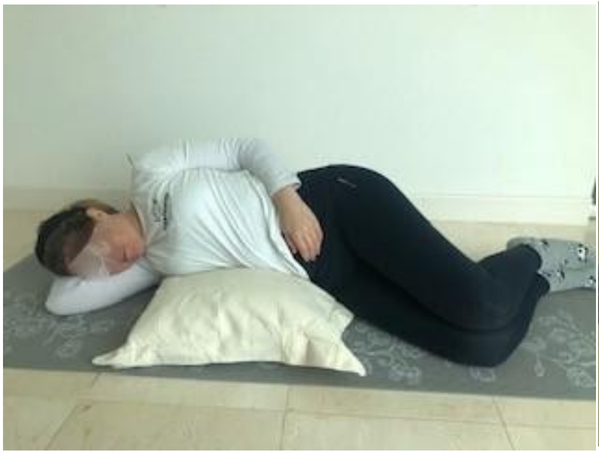

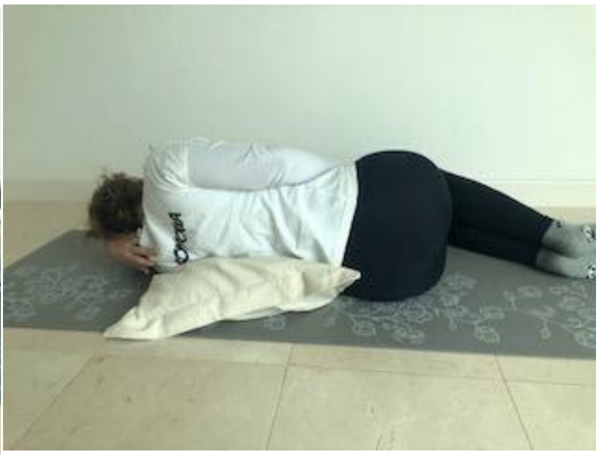

- EXERCISE 4: Reeducation of the anterior region of the diaphragm

The patient will be layed in prone position with a pillow between pelvic región and abdomen. Hands will be placed under the head, with the head rotated. The exercise starts with a slight expiration with abdominal contraction at the end, followed by a slow and prolonged inspiratory phase

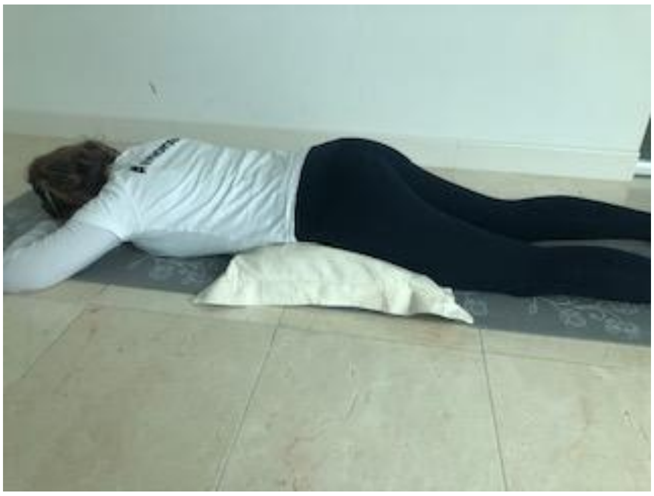

- EXERCISE 5: Reeducation of the anterior region of the diaphragm

The patient will be in a 4 point kneeling position doing the same breathing phases. At first, the patient will perform an expiration with pursed lips until produce an abdominal wall contraction. Later, the patient will perform a deep nasal inspiration with the mouth closed. With this maneuver, the aim is to bring the air to the belly; when plenty of aire, the aire will be expulsed while the abdomen is contracted. 10-15 repetitions in the morning and 10-15 in the evening.

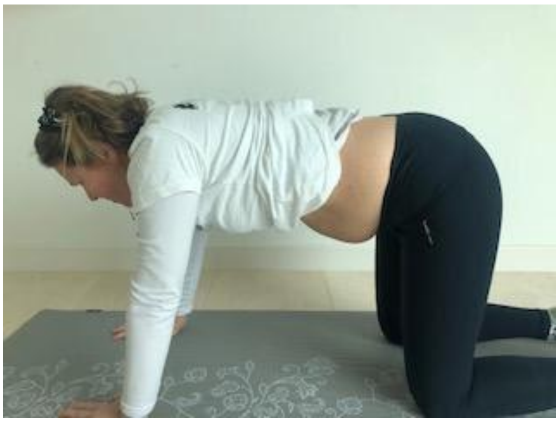

.

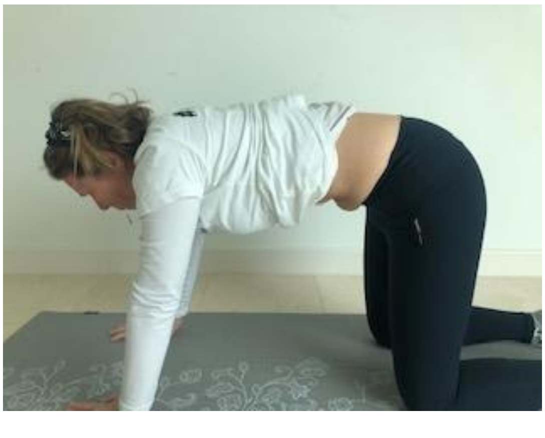

- EXERCISE 6: Exercise of positive expiratory pressure

Through this exercise, an expiratory brake is performed, what improves bronchial stability and prevents collapse. A delay in the air outlet occurs, maintining a distension in upper aire passages. It favors the opening of colateral ventilation, what makes the air to reach peripheral areas that can not be reached because of obstruction. It improves the elimination of secretions in a secondary way[15]. Through a home made device, the patient will perform a deep inspiration and, following that, a slow expiration blowing through the tube introduced in a bottle of water, not achieving a complete expiration. The secuence will be repeated 10-15 times, twice a day.

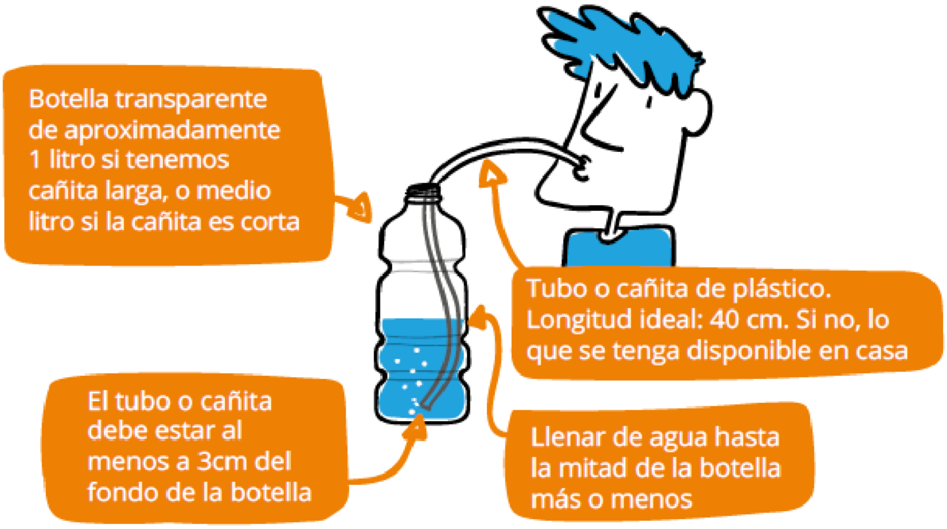

Picture from College of Physical Therapists, Community of Madrid

- EXERCISE 7: costal breathing exercise in supine position

The patient will be layed in supine position over a hard surface with a pillow under the head and knees. An increase in inspiration and expiration volumen will be performed. The patient will place a hand on the abdomen, and it will work as a brake. For the abdomen. At first, an inspiration with mouth closed will be done, and the air will arrive to lungs. Later, with pursed lips, the patient will empty the lungs in a forced expiration.

It is important the hand's rol, because the abdomen should not raise, to trigger a greater activation of itercostal muscles. 10-15 repetitions, twice a day

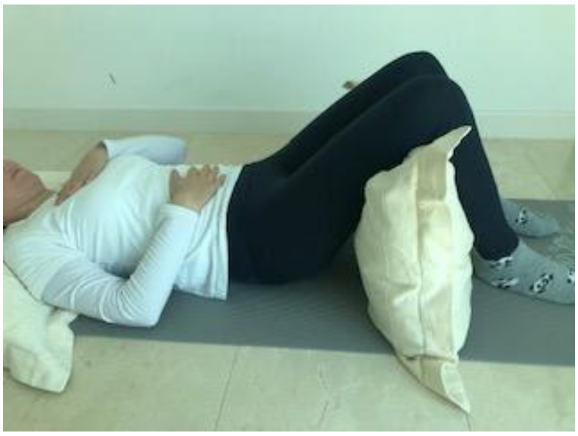

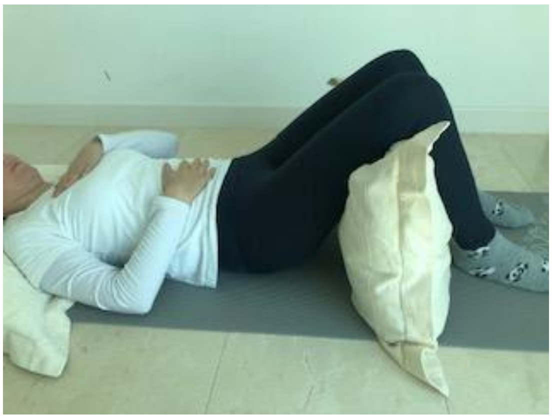

- EXERCISE 8. Costal breathing exercise in right lateral decubite

The patient will be layed in lateral decubite over the right side. This exercise aims to work expansion and mobility of left hemithorax. A pillow will be placed under the right hemithorax to block right thoracic wall. Left arm will be stretched over the head to increase the thoracic expansion. At first, an inspiration with mouth closed will be performed. The air will arrive to lungs, and then, with pursed lips, the patient will empty the lungs in a forced expiration.10-15 repetitions, twice a day.

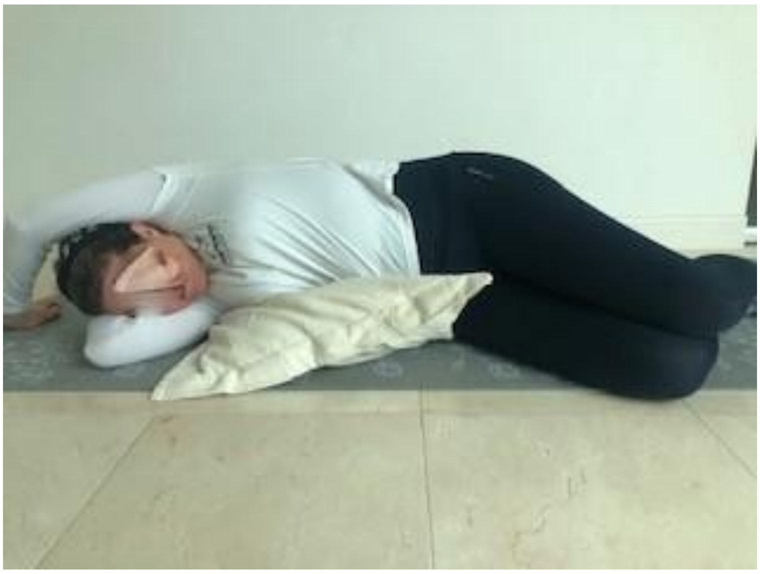

- EXERCISE 9. Costal breathing exercise in left lateral decubite

The patient will be layed in lateral decubite over the left side. This exercise aims to work expansion and mobility of right hemithorax. A pillow will be placed under the right hemithorax to block right thoracic wall. Left arm will be stretched over the head to increase the thoracic expansion. At first, an inspiration with mouth closed will be performed. The air will arrive to lungs, and then, with pursed lips, the patient will empty the lungs in a forced expiration.10-15 repetitions, twice a day.

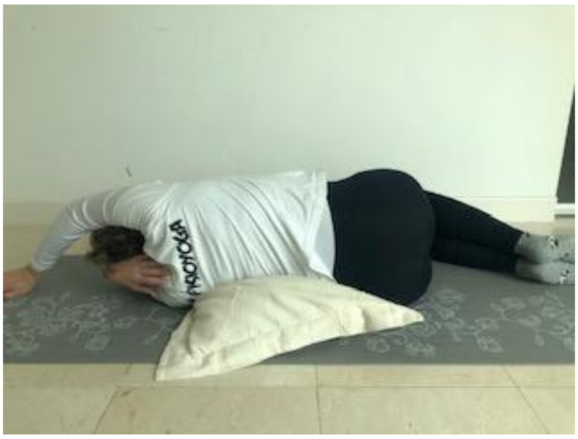

- EXERCISE 10. Costal reeducation exercise in sitting position

The patient will be placed in a sitting position with knees and hips in 90º. Hands will be placed over low ribs. At first, an inspiration with pursed lips will be performed. The patient must feel the aire under the hands, without making resistance. 1-2 seconds of apnea are developed, and, pursing lips, the air will be slowly expired. Hands will help in this process by making pressure over ribs. 10-15 repetitions, twice a day.

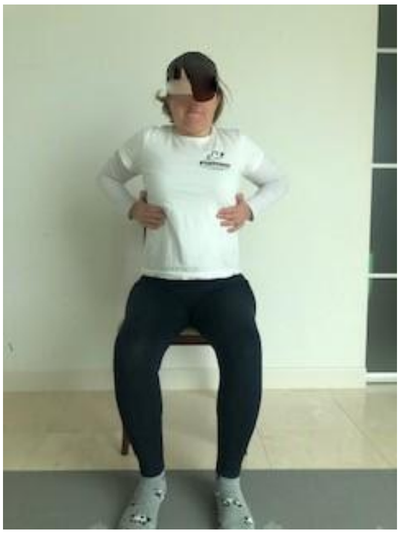

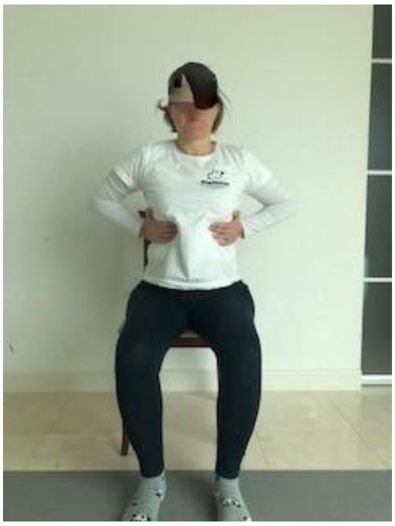

Non-specific Toning Exercise Program

Home Exercises. COVID19

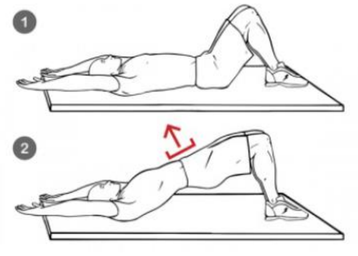

3 Series **12** reps
Hold the bridge up for 4 seconds

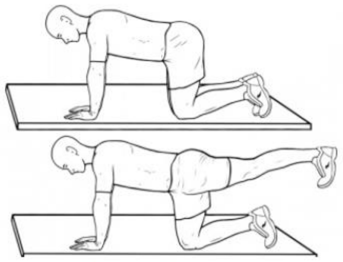

3 Series **12** reps
Maintain the position for 4 seconds, for each repetition and leg

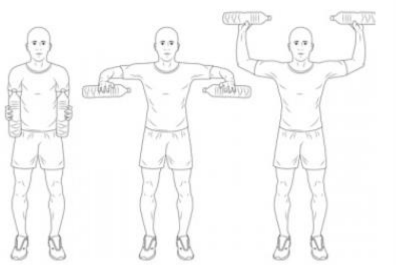

3 Series **12** reps

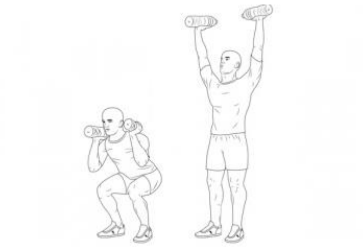

3 Series **12** reps

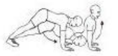

3 Series **12** reps

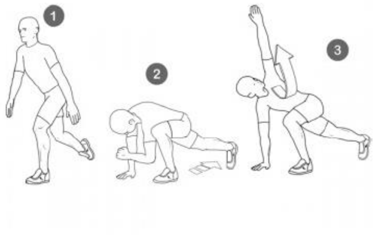

3 Series **12** reps

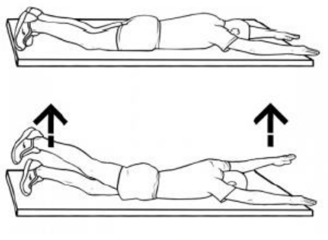

3 Series **12** reps
Maintain the position for 4 seconds

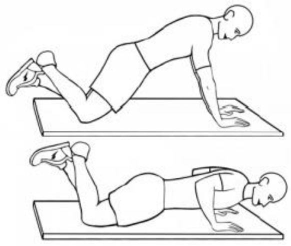

3 Series **12** reps

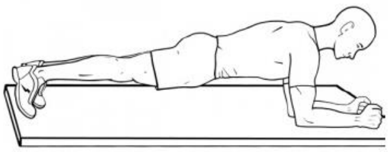

3 Series **30** seg **1** min desc

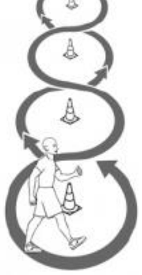

3 Series **180** seg **2** min desc
