# Supplementary material for: Therapeutic pulmonary telerehabilitation protocol for patients affected by COVID-19, confined to their homes: study protocol for a randomized controlled trial
Source: Trials. 2020 Jun 29;21:588. doi: 10.1186/s13063-020-04494-w (PMC7322707; doi:10.1186/s13063-020-04494-w)
Supplement: Supplementary file 2 — Additional file 2. Appendix 2. Checklist for telephone monitoring. [file 13063_2020_4494_MOESM2_ESM.docx]

**Appendix 1. Checklist for telephone monitoring**

| **Checklist for clinical evaluation during telephone follow-up of home care** | | |
| --- | --- | --- |
| Compared to last day we contacted with you, how do you feel today?  *Better*  *Same*  *Worst* | | |
| **QUESTIONS** | Yes | No |
| Temperature | | |
| · High fever ( ≥ 39ºC) |  |  |
| Cough | | |
| · Persistent cough that makes you difficult to eat and hydrate |  |  |
| Severe dyspnea | | |
| · Fatigue when getting up and starting to walk or at rest |  |  |
| · Duration of non-severe respiratory distress beyond 10 days |  |  |
| Tachypnea: broken speech or difficulty holding the conversation |  |  |
| Inability to carry out any type of physical activity |  |  |
| Pleuritic type chest pain | | |
| · Rib pain that makes it hard to breathe, new or worsening to previous pain |  |  |
| Hemoptisis |  |  |
| Alertness (if any doubts in the interview with the patient, ask another person who is currently with the patient) | | |
| · Aware and alert |  |  |
| · Answer questions normally |  |  |
| Uncontrollable vomiting that prevents you from feeding |  |  |
| Abundant diarrhea (≥ 10 bowel movements in one day or between 5-10 for more than 3 days) |  |  |
| After 7th day, the following should be considered: | | |
| · Fever duration (≥ 38 ºC) greater than 7 days |  |  |
| · Cough duration greater than 14 days |  |  |
| Psychosocial conditions of home isolation have changed |  |  |
